# Magnetic Hyperthermia in Y79 Retinoblastoma and ARPE-19 Retinal Epithelial Cells: Tumor Selective Apoptotic Activity of Iron Oxide Nanoparticle

**DOI:** 10.1167/tvst.8.5.18

**Published:** 2019-09-27

**Authors:** Hakan Demirci, Naziha Slimani, Mercy Pawar, Ronald E. Kumon, Prem Vaishnava, Cagri G. Besirli

**Affiliations:** 1 Department of Ophthalmology and Visual Sciences. W. K. Kellogg Eye Center, University of Michigan, Ann Arbor, MI, USA; 2 Department of Physics, Kettering University, Flint, MI, USA

**Keywords:** magnetic hyperthermia, nanoparticle, dextran-coated iron oxide nanoparticle, retinoblastoma, retina pigment epithelium

## Abstract

**Purpose:**

To evaluate selective apoptosis of Y79 retinoblastoma versus ARPE-19 retinal pigment epithelial cells by using different doses of dextran-coated iron oxide nanoparticles (DCIONs) in a magnetic hyperthermia paradigm.

**Methods:**

Y79 and ARPE-19 cells were exposed to different concentrations of DCIONs, namely, 0.25, 0.5, 0.75, and 1 mg/ml. After 2 hours of incubation, cells were exposed to a magnetic field with a frequency of 250 kHz and an amplitude of 4 kA/m for 30 minutes to raise the cellular temperature between 42 and 46°C. Y79 and ARPE-19 cells incubated with DCION without magnetic field exposure were used as controls. Cell viability and apoptosis were assessed at 4, 24, and 72 hours after hyperthermia treatment.

**Results:**

At 4 hours following magnetic hyperthermia, cell death for Y79 cells was 1%, 8%, 17%, and 17% for 0.25, 0.5, 0.75 and 1 mg/ml of DCION, respectively. Cell death increased to 47%, 59%, 70%, and 75% at 24 hours and 16%, 45%, 50%, and 56% at 72 hours for 0.25, 0.5, 0.75, and 1 mg/ml of DCIONs, respectively. Magnetic hyperthermia did not have any significant toxic effects on ARPE-19 cells at all DCION concentrations, and minimal baseline cytotoxicity of DCIONs on Y79 and ARPE-19 cells was observed without magnetic field activation. Gene expression profiling showed that genes involved in FAS and tumor necrosis factor alpha signaling pathways were activated in Y79 cells following hyperthermia. Caspase 3/7 activity in Y79 cells increased following treatment, consistent with the activation of caspase-mediated apoptosis and loss of cell viability by magnetic hyperthermia.

**Conclusion:**

Magnetic hyperthermia using DCIONs selectively kills Y79 cells at 0.5 mg/ml or higher concentrations via the activation of apoptotic pathways.

**Translational Relevance:**

Magnetic hyperthermia using DCIONs might play a role in targeted management of retinoblastoma.

## Introduction

Retinoblastoma is one of the most common malignant intraocular tumors in children. In the United States, it is estimated that about 12 children per million under the age of 5 years are affected by this cancer.^1^ In recent years, significant progress has been made in the treatment of retinoblastoma with the introduction of intra-arterial and intravitreal chemotherapy, and as a result, the survival rate reached above 90% in developed countries.^2^,^3^ A recent literature review showed that the globe salvage rate is 90% to 100% for groups A to C eyes, more than 70% for group D eyes, and 51% for group E eyes following the intra-arterial chemotherapy. The primary reason for treatment failure in groups D and E was the presence of intravitreous seeds. Intra-arterial chemotherapy requires an experienced team and advanced equipment, which are limited to few centers.^4^,^5^ With the recent development of safe injection techniques, intravitreal injections of the chemotherapeutic agents melphalan and topotecan are being used more commonly. However, intravitreal chemotherapy has the potential to cause ocular toxicity. Therefore, there is an unmet need for new retinoblastoma treatment modalities with a better safety and efficacy profile.

Nanoparticles of various materials can enhance intraocular drug delivery and retention and may have the potential to improve retinoblastoma treatment.^6^,^7^ Most systems used for drug delivery applications are colloidal suspensions consisting of nanoparticles with a diameter ranging from 10 to 1000 nm, in which the therapeutic agent can be encapsulated or conjugated on the surface of the nanoparticles. Mitra et al.^8^ demonstrated that etoposide-loaded poly(lactide-co-glycolide) nanoparticles show 100 times greater antiproliferative and apoptotic gene activity in Y79 retinoblastoma cells compared to native etoposide, indicating the potential of a nanoparticle platform as novel chemotherapy delivery system for retinoblastoma. Similarly, Ahmed et al.^9^ reported that carboplatin-loaded protein nanoparticles had greater intracellular uptake and sustained retention, increasing the antiproliferative activity on retinoblastoma cells compared to their soluble counterpart. Qu et al.^2^ studied the efficacy of topotecan-loaded mesoporous silica nanoparticles that were surface conjugated with folic acid for the treatment of retinoblastoma. These nanoparticles showed a remarkable uptake in cells compared to the nontargeted nanoparticles, and higher cytotoxicity was observed on retinoblastoma cells. Chitosan nanoparticles, which are biocompatible, relatively nontoxic, and biodegradable in nature,^10^ have been used by some researchers due to their improved mucoadhesiveness and ability to cross the epithelial junction. Manasi Das et al.^11^ encapsulated anticancer drug nutlin-3a and chemosensitizer curcumin in polylactic-co-glycolic acid nanoparticles, which were surface functionalized with folate to enhance the therapeutic potential and prevent multidrug resistance. Beside polylactic-co-glycolic acid nanoparticles, lipid nanoparticles have been used to codeliver miR-181a and melphalan to retinoblastoma cell lines to decrease the expression of the antiapoptotic gene BCL2 while increasing the expression of proapoptotic gene BAX, thus making the cells more sensitive to melphalan treatment.^12^

Magnetic nanoparticles have recently emerged as a potential modality for the diagnosis and the treatment of cancer.^13^ Super paramagnetic nanoparticles of iron oxide have proven particularly beneficial for potential cancer treatment. These materials are also called multifunctional ferrofluids, which are highly stable in aqueous solutions and have been tested for the delivery of hydrophilic and hydrophobic drugs. A ferrofluid that contains super paramagnetic iron oxide nanoparticles can be functionated with many entities, such as an imaging agent and a drug to treat cancer in one unit. This multifunctional unit possesses a unique ability to generate heat when exposed to an alternating magnetic field. Such an ability has a potential application of not only delivering heat to kill cancer cells but also delivering a payload of drug to the tumor, increasing the efficacy of the treatment.^14^

Heat, or hyperthermia, as therapeutic tool has been used for the management of cancer for many years. Previous studies have shown that tumor cells are more sensitive to heat than normal cells.^15^–^18^ Sustained temperatures above 43°C sensitize tumor cells to respond favorably to radiotherapy or chemotherapy and produce a direct cytotoxic effect.^15^,^16^,^18^ Over the years, the methods of heat application have improved from water baths to noncontact heat-delivery techniques, including radiofrequency, microwaves, and focused ultrasound waves. Recently, magnetic nanoparticles have been used as nanoheaters in the delivery of heat to targeted tumor cells without damaging normal tissue. This phenomenon is commonly known as magnetic hyperthermia.^18^ Magnetic nanoparticles typically have a diameter of 2 to 100 nm and can be coated with a biocompatible substance to prevent agglomeration. In magnetic hyperthermia, the application of an alternating magnetic field to the magnetic nanoparticles leads to the coupling of magnetic motions of the nanoparticles with the oscillating field and conversion of the absorbed energy into heat within the targeted tumor tissue. Three distinct mechanisms are involved in heat generation by nanoparticles: Neel relaxation (release of energy by the rotation of the magnetic moment of the nanoparticles), Brownian relaxation (release of the energy by physical rotation of the nanoparticles), and hysteresis loses (when the magnetism of nanoparticles does not follow the magnetism of the applied field).^17^,^18^

Hyperthermia was previously used in combination with external beam radiotherapy in the management of uveal melanoma and retinoblastoma.^19^ In this paradigm, local heat is generated via ultrasound and microwave probes. Finger^20^ utilized a dish-shaped microwave antenna that was placed over the sclera under the tumor at the time of plaque radiotherapy. This antenna delivered heat at a minimum of 42°C for 45 minutes. Hyperthermia in combination with plaque radiotherapy (thermoradiotherapy) reduced the tumor apex dose from 100 Gy to a mean of 53 Gy and provided a local control rate of 97% at a mean follow-up of 45 months. In a murine transgenic retinoblastoma model, Murray et al.^21^ tested two ferromagnetic hyperthermia needles placed into the medial and lateral canthi abutting the globe. After applying heat for 20 minutes at 48°C, retinoblastoma was cured in 30% of eyes. When the heat increased to 54°C, the cure rate improved to 100%. Additionally, external beam radiation dose for tumor control in 50% of eyes decreased from 27.6 Gy to 3.3 Gy when combined with magnetic hyperthermia.

Nanoparticles activated by magnetic hyperthermia can produce reactive oxidative species that lead to DNA and mitochondrial damage and protein oxidation.^22^–^28^ In addition, the activation of the phosphatidylinositol 3-kinase/Akt/Bad pathway by magnetic hyperthermia potentiates the intrinsic proapoptotic factors cytochrome c, caspase 9, and caspase 3, causing apoptotic death.^22^–^29^ Exposure to a mild heat shock can activate extrinsic apoptotic signaling via FAS signaling in cancer cell lines, including Jurkat and HeLa cells.^25^ Besides the FAS death receptor, magnetic nanoparticles have been shown to induce cell death in human glioma cells via increased tumor necrosis factor alpha (TNF-α) expression.^28^

Magnetic nanoparticles as nanoheaters that can deliver targeted heat to tumor tissue have not been previously evaluated in the management of retinoblastoma. With the unique superficial location of the eye and the advancement of safe intravitreal injection techniques, magnetic nanoparticles can easily be used for drug delivery and enhance the effect of current chemotherapeutics. In this study, we assessed the apoptotic effect of dextran-coated iron oxide nanoparticles (DCIONs) activated by a magnetic field on a Y79 retinoblastoma cell line. We evaluated the time course of cell death and the signaling pathways activated during magnetic hyperthermia of Y79 cells. We compared Y79 cell line responses to those seen in ARPE-19 cell line, an in vitro model of retinal pigment epithelium (RPE) cells, as a proxy for one of the retinal cell types that might be adversely affected by this treatment when delivered intravitreally.

## Methods

### Cell Culture

The Y79 retinoblastoma cell line was maintained as a suspension culture in RPMI 1640 media (catalog number 30-2001; Invitrogen, Waltham, MA) with 2 mM L-glutamine, 20% heat-inactivated fetal bovine serum (FBS), 100 U/ml penicillin, and 100 μg/ml streptomycin. The ARPE-19 retinal pigment epithelium cell line was cultured in 1:1 DMEM:F12 media (catalog number 30-2006; Invitrogen) containing 2.5 mM L-glutamine, 15 mM HEPES, 10% FBS, 100 U/ml penicillin, and 100 μg/ml streptomycin. Both cells lines were plated on 75-cm^2^ flasks (catalog number 07-202-000; Fisher Scientific, Ann Arbor, MI) and maintained at 37°C in a humidified atmosphere of 5% CO_2_ and 95% air until they reached 90% confluency.

### Magnetic Hyperthermia

Iron oxide nanoparticles were synthesized using a standard coprecipitation technique in which an aqueous solution of FeCl_3_·6H_2_O and FeCl_2_·4H_2_O were mixed in a 2:1 molar ratio and Fe_3_O_4_ nanoparticles were precipitated by the drop-wise addition of 1M NH_4_OH. During precipitation, N_2_ gas was bubbled through the solution to protect against oxidation of the Fe^2+^ ions into Fe^3+^ ions. The precipitate was separated from the solution by a strong magnet, washed with deionized water and resuspended in a metastable 0.5 M NaOH solution. In order to suspend the precipitated nanoparticles in a carrier solution (deionized water), they were coated in dextran by the drop-by-drop addition of the metastable solution of Fe_3_O_4_ to a solution of 15 to 20 kDa dextran (MP Biomedicals, Santa Ana, CA) in 0.5 M NaOH while simultaneously probe sonicating.

Y79 retinoblastoma and ARPE-19 cells were cultured in cryogenic tubes (catalog number 03-337-7D; Fisher Scientific) at a density of 100,000 cells/ml. They were incubated for 24 hours prior to treatment. DCION stock was diluted with the respective culture media to give a dose of 0.25, 0.5, 0.75, or 1 mg/ml. After removing the media, cells were treated for 2 hours to allow receptor-mediated endocytosis (clathrin- and caveolae-mediated endocytosis) in the dose-dependent concentration of nanoparticles, as shown in multiple previous studies.^29^,^30^ After 2 hours of incubation, media with nanoparticles were removed and fresh media were added. Cells were then treated using a magnetic field with the frequency and intensity values of 250 kHz and 4 kA/m. After reaching a temperature varying between 42 and 46°C, the electromagnetic gradient was kept constant for 30 minutes. Temperature was measured using a fiber optic thermometer (Optocon AG, Dresden, Germany). Controls were untreated Y79 and ARPE-19 cells and those incubated with respective concentrations of nanoparticles without magnetic hyperthermia.

### Cell Viability and Caspase Activity Assays

Hyperthermia-induced apoptosis was assessed at 4, 24, and 72 hours after treatment for both Y79 and ARPE-19 cells. The numbers of viable cells were measured based on quantitation of adenosine triphosphate by using the CellTiter-Glo Luminescent Cell Viability Assay kit (catalog number G7571; Promega, Madison, WI) following the manufacturer's recommendations. Caspase 8 or 3/7 activity of Y79 cells at the nanoparticle concentration of 1 mg/ml was measured according to manufacturer's instructions after 48 hours of treatment by using the Caspase-Glo 8 or 3/7 Assay kit (Promega). Luminescence was measured using a Veritas Microplate luminometer (Turner Biosystems, Sunnyvale, CA).

### Transmission Electron Microscopy (TEM) Imaging of DCION Uptake

TEM was performed to elucidate the cellular uptake and distribution of nanoparticles. Y79 and ARPE-19 cells were cultured in Corning 100-mm plates (catalog number 430167; Fisher Scientific) for 24 hours, after which they were incubated with 1 mg/ml of nanoparticles for 2 hours. After treatment, cells were collected and fixed in 2.5% glutaraldehyde and subsequently postfixed using 1% osmium tetroxide for 3 hours, and then pelleted, dehydrated, infiltrated, and embedded. Finally, ultrathin sections were cut and stained with uranyl acetate. TEM images were taken using a Hitachi HT7700 transmission electron microscope (Tokyo, Japan).

### PCR Assay for Apoptosis Gene Profiling

Y79 retinoblastoma cells were treated with 1 mg/ml of DCION followed by the induction of a magnetic field to create hyperthermia. The cells were further incubated for 24 hours after magnetic induction. Total RNA was extracted using the RNeasy mini kit (catalog number 74104; Qiagen, Germantown, MD). A total of 25 ng of complementary DNA (cDNA) was then synthesized and preamplified using the RT^2^ PreAMP cDNA Synthesis kit (catalog number 330451; Qiagen) following the manufacturer's instructions. A solution of 25 μl made of the amplified cDNA diluted with nuclease-free water and RT^2^ SYBR Green qPCR Mastermix (catalog number 330502; Qiagen) was added to each well of the RT^2^ Profiler PCR Array Human Apoptosis (catalog number PAHS-012Z; Qiagen) plate. Real-time PCR was performed on a CFX96 PCR system (Bio-Rad Laboratories, Hercules, CA) by using SYBR green detection. The thermocycler parameters consisted of an initial denaturation at 95°C for 10 minutes followed by 39 cycles at 95°C for 15 seconds and annealing at 60°C for 1 minute. The dissociation curve was at 95°C for 1 minute, 55°C for 30 seconds, and 95°C for 30 seconds. All quality-control requirements stated by the manufacturer were fulfilled in the PCR runs (including genomic DNA control, cDNA synthesis control, and positive PCR controls). Relative changes in gene expression were calculated using the delta delta cycle threshold (ΔΔCt) method using SA Bioscience's PCR Array Data Analysis Web Portal. Gene set enhancement analysis was performed by using web tools, including www.reactome.org and toppgene.cchmc.org. The pathways found to be statistically significant were reported.

### Data Analysis

Data were analyzed using one-way analysis of variance followed by Tukey's post hoc test. A value of *P* < 0.05 was considered significant. Prism 7.0 (GraphPad Software, San Diego, CA) was used for all statistical analyses. Results are expressed as mean ± standard error of the mean.

## Results

**Figure 1:**
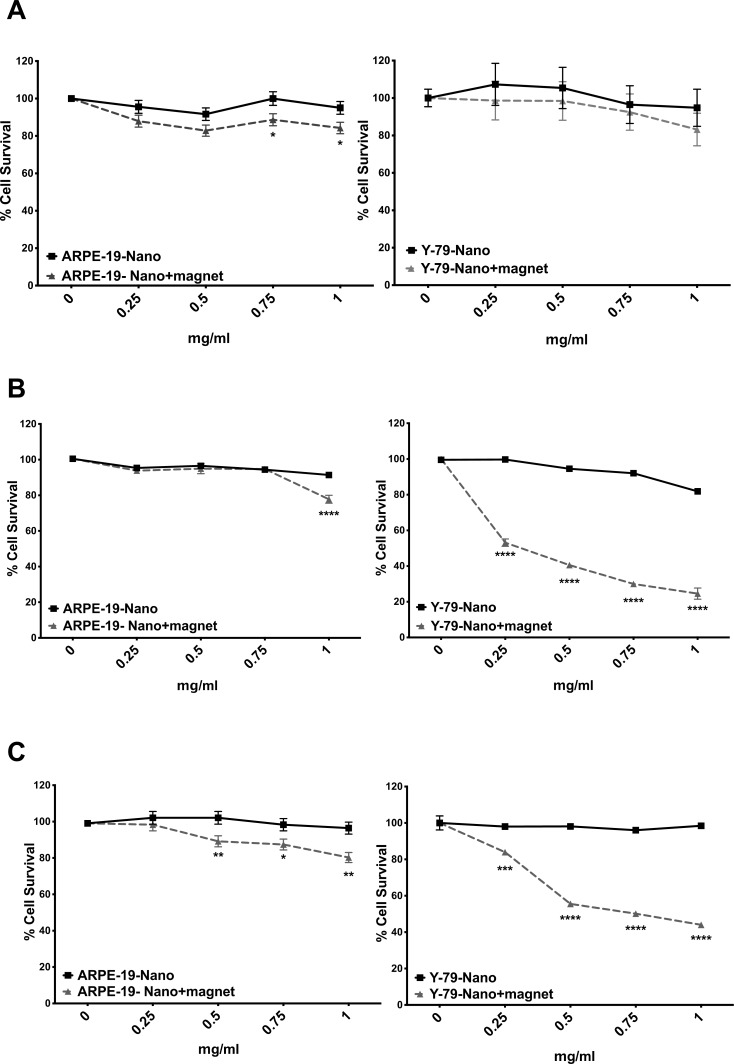
ARPE-19 and Y79 retinoblastoma cells were treated with iron oxide nanoparticles, and viability was measured with or without magnetic hyperthermia at 4 (A), 24 (B), and 72 (C) hours. n = 3, mean ± SEM, *P < 0.05, **P < 0.005, ***P < 0.0005, ****P < 0.0001 for each time point, Tukey test, N = 3.

We first tested the viability of Y79 retinoblastoma and ARPE-19 RPE cell lines by using different DCION concentrations at 4, 24, and 72 hours following magnetic hyperthermia treatment (Fig. 1). A time- and concentration-dependent cell death was observed in Y79 cells after the application of alternating electromagnetic field. At 4 hours following magnetic hyperthermia, cell death for Y79 cells was 1%, 8%, 17%, and 17% for 0.25, 0.5, 0.75, and 1 mg/ml of DCION concentration, respectively. At 24 hours, cell death in Y79 cells was 47%, 59%, 70%, and 75%, and at 72 hours, cell death was 16%, 45%, 50%, and 56% at DCION concentrations of 0.25, 0.5, 0.75, and 1 mg/ml, respectively. More than half of cells were dead when exposed to 0.75 and 1 mg/ml of DCION concentrations after 24 and 72 hours. In contrast, ARPE-19 cells remained viable up to 88% at 0.25, 0.5, and 0.75 mg/ml of DCION concentrations following magnetic hyperthermia after 24 and 72 hours. There was minimal cytotoxicity of iron oxide nanoparticles to Y79 or ARPE-19 cells for all concentrations tested in the absence of magnetic hyperthermia (Fig. 1).

**Figure 2:**
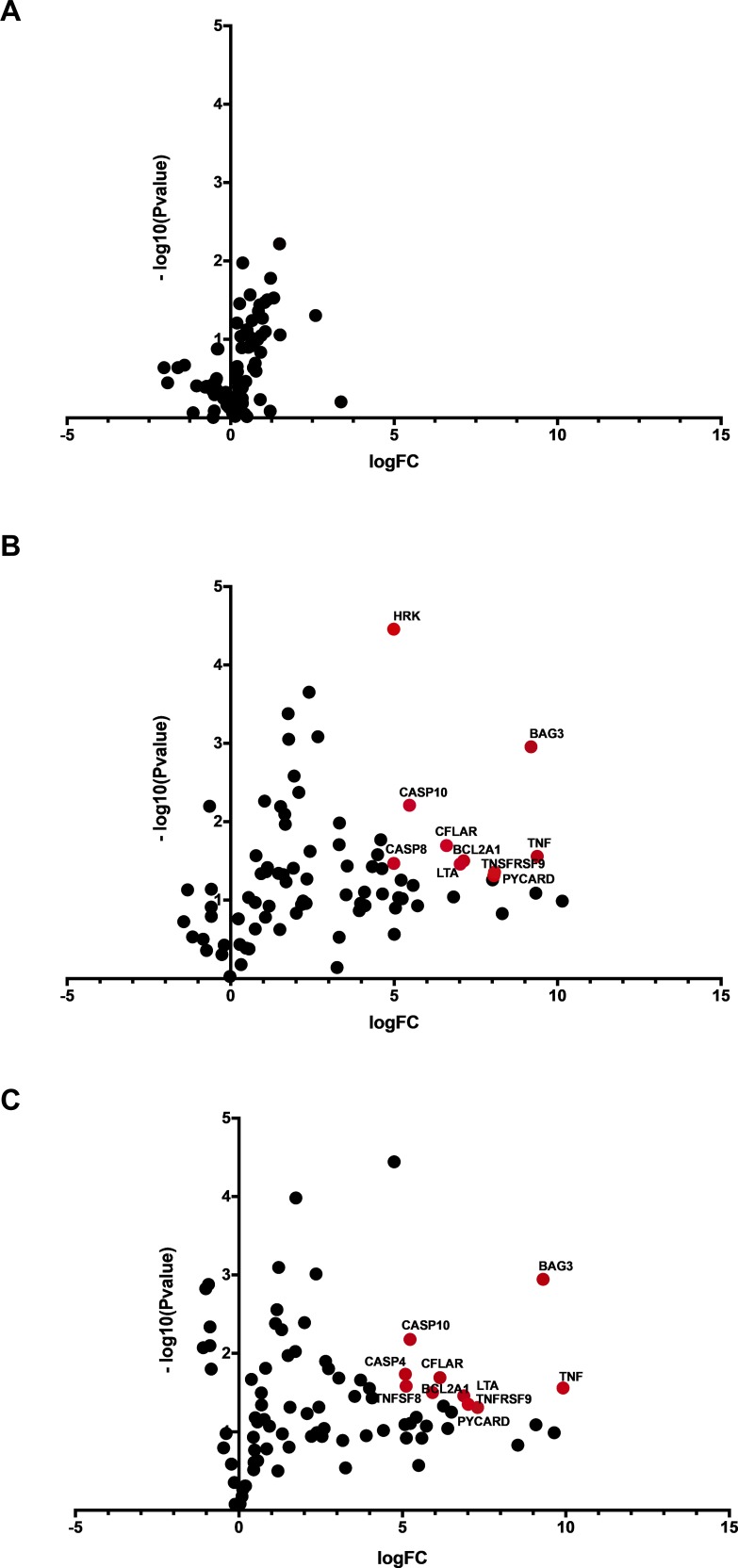
Volcano plot of gene expression profiling showing Y79 retinoblastoma cells exposed to iron oxide nanoparticles versus untreated Y79 retinoblastoma cells (A), Y79 retinoblastoma cells exposed to magnetic hyperthermia using iron oxide nanoparticles versus Y79 retinoblastoma cells without iron oxide nanoparticles exposure (B), Y79 retinoblastoma cells exposed to magnetic hyperthermia using iron oxide nanoparticles versus Y79 retinoblastoma cells exposed to iron oxide nanoparticles without magnetic field exposure (C). Genes with a 30-fold or larger increase in messenger RNA (mRNA) levels with a P value of <0.05 are indicated in red and names are shown. FC, Fold change.

We evaluated the expression of genes involved in cell death pathways 24 hours after magnetic hyperthermia in Y79 retinoblastoma cells (Fig. 2). Gene set enhancement analysis confirmed the activation of several genes involved in cell death mediated by death receptor signaling, including FAS and TNF-α pathways. *FASLG*, *TNF*, *TNFRSF9*, and *TNFRSF1A*, genes involved in FAS and TNF pathways, were expressed at significantly higher levels in Y79 retinoblastoma cells treated with magnetic hyperthermia compared to untreated cells (Table 1). We also measured the effect of DCION exposure alone on gene expression by comparing Y79 retinoblastoma cells treated with or without magnetic hyperthermia. Again, we found that the levels of *FASLG*, *TNF*, *TNFRSF9*, and *TNFRSF1A* were significantly elevated after magnetic hyperthermia in Y79 cells when compared to cells exposed to DCION alone (Table 2). Our data demonstrate a global increase in cell death gene expression after magnetic hyperthermia in retinoblastoma cells. This activation leads to a major shift in apoptotic signaling pathways and leads to increased cell death, consistent with the loss of cell viability seen specifically in Y79 cells exposed to DCION-mediated hyperthermia (Fig. 1). Tables 1 and 2 include the top 25 genes seen in our expression profiling experiments. The full list of genes that respond to magnetic hyperthermia after DCION treatment is presented in supplemental Tables 1 and 2.

**Table 1:** Top 25 Genes Upregulated 24 Hours After Magnetic Hyperthermia in Y-79 Retinoblastoma Cells Treated With 1 mg/ml of DCIONs Compared to Untreated Cells

Gene	Description	Fold Change
*FASLG*	FAS ligand	1133.0533
*TNF*	Tumor necrosis factor	665.9792
*IL10*	Interleukin 10	646.273
*BAG3*	BCL2 associated athanogene 3	583.8012
*CASP14*	Caspase 14	317.2186
*TNFRSF9*	TNF receptor superfamily member 9	269.2246
*PYCARD*	PYD and CARD domain containing	265.5181
*LTBR*	Lymphotoxin beta receptor	258.2575
*BCL2A1*	BCL2 related protein A1	140.0049
*LTA*	Lymphotoxin alpha	130.0269
*BIRC3*	Baculoviral IAP repeat containing 3	112.9338
*CFLAR*	CASP8 and FAAD-like apoptosis regulator	97.4101
*CD70*	CD70 molecule	52.6855
*CD40*	CD40 molecule	47.9237
*CASP10*	Caspase 10	44.4055
*BCL2L10*	BCL2 like 10	38.037
*CD40LG*	CD40 ligand	37.1683
*CASP5*	Caspase 5	35.2447
*TNFRSF1A*	TNF receptor superfamily member 1A	32.9605
*FAS*	FAS cell surface death receptor	32.1334
*CASP8*	Caspase 8	31.8378
*HRK*	Harakiri, BCL2 interacting protein	31.7643
*CD27*	CD27 molecule	25.0372
*TNFSF10*	TNF superfamily member 10	24.7496
*CASP4*	Caspase 4	24.1285

**Table 2:** Top 25 Genes Upregulated 24 Hours After Magnetic Hyperthermia in Y-79 Retinoblastoma Cells Treated With 1 mg/ml of DCIONs Compared to Y-79 Retinoblastoma Cells Exposed to DCIONs Without Magnetic Field Application

Gene	Description	Fold Change
*TNF*	Tumor necrosis factor	964.3592
*FASLG*	FAS ligand	800.8011
*BAG3*	BCL2 associated athanogene 3	632.7203
*IL10*	Interleukin 10	543.3515
*CASP14*	Caspase 14	369.8034
*PYCARD*	PYD and CARD domain containing	157.6189
*TNFRSF9*	TNF receptor superfamily member 9	129.4063
*LTA*	Lymphotoxin alpha	117.7604
*LTBR*	Lymphotoxin beta receptor	90.524
*BIRC3*	Baculoviral IAP repeat containing 3	83.6729
*CD40LG*	CD40 ligand	76.4886
*CFLAR*	CASP8 and FADD like apoptosis regulator	70.8628
*BCL2A1*	BCL2 related protein A1	60.5563
*BCL2L10*	BCL2 like 10	53.2101
*TNFRSF1A*	TNF receptor superfamily member 1A	48.284
*FAS*	FAS cell surface death receptor	45.187
*CD40*	CD40 molecule	42.9574
*RIPK2*	Receptor interacting serine/threonine kinase 2	37.947
*CASP10*	Caspase 10	37.6304
*CD70*	CD70 molecule	34.8205
*TNFSF8*	TNF superfamily member 8	34.6293
*CASP4*	Caspase 4	34.1119
*CD27*	CD27 molecule	33.7515
*HRK*	Harakiri, BCL2 interacting protein	26.8848
*TNFRSF10A*	TNF receptor superfamily member 10a	21.4137

**Figure 3:**
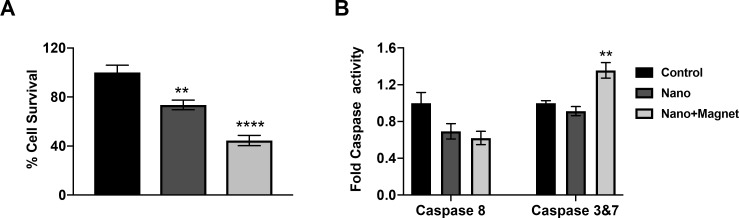
Y79 cell viability (A) and caspase activity (B) 48 hours following magnetic hyperthermia using iron oxide nanoparticles at a concentration of 1 mg/mL. n = 3, mean ± SEM, **P < 0.005, ****P < 0.0001 compared to control, Tukey test, N = 3.

To confirm the activation of apoptosis, we evaluated caspase 8 and 3/7 activities at 48 hours after magnetic hyperthermia. Caspase 3/7 activity levels were significantly elevated in Y79 cells treated with the magnetic hyperthermia compared to control cells or cells treated with magnetic nanoparticles alone (Fig. 3). In contrast, caspase 8 activity did not increase in Y79 cells after magnetic hyperthermia (Fig. 3). This suggests that the intrinsic apoptotic pathway mediated by mitochondria is the primary cell death mechanism in Y79 cells following hyperthermic insult induced by magnetic nanoparticles.

**Figure 4:**
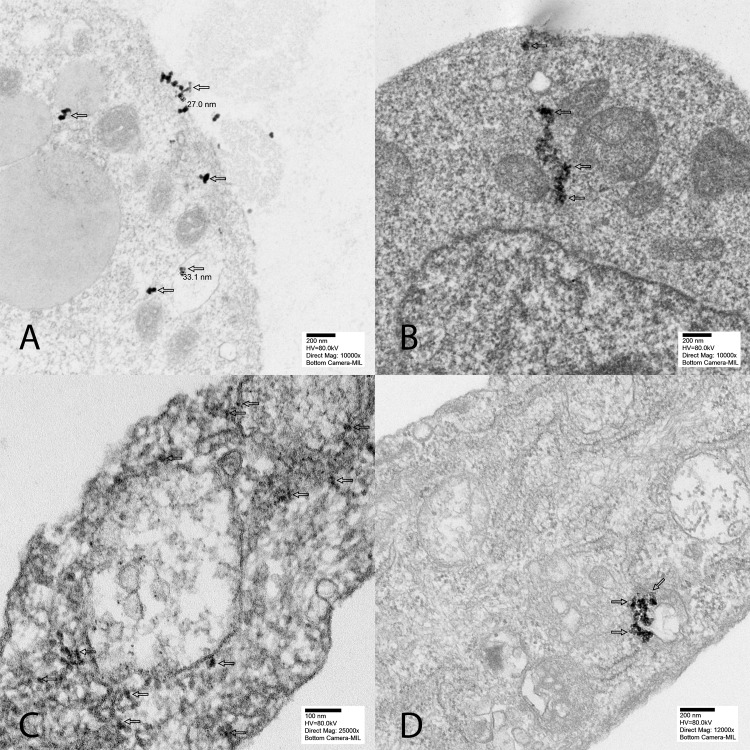
Electron microscopy showed that nanoparticles are engulfed by both ARPE-19 and Y79 cells. The magnetic nanoparticles are located in endosomes and lysosomes throughout the cytoplasm in ARPE-19 cells (arrows, A and B) and Y79 retinoblastoma cells (arrows, C and D).

We next evaluated the localization of DCIONs in Y79 and ARPE-19 cells using TEM. Our data show that nanoparticles were engulfed by both Y79 and ARPE-19 cells (Fig. 4). In ARPE-19 cells, DCIONs were located in lysosomes and endosomes in the cytoplasm, whereas nanoparticles were distributed throughout cellular structures in Y79 cells.

## Discussion

Previous studies have shown that 43°C is a critical temperature for the survival of normal and cancer cells. A review of the literature by Leith et al.^22^ demonstrated that cell survival decreases exponentially as a function of exposure time at temperatures above 41°C in multiple cancer cell lines, including HeLa, L1210 leukemia, EMT6 mouse mammary sarcoma, and malignant liver cells.^22^–^24^ Additionally, at the temperatures 43°C or above, cell death increased by a factor of 2 for every 1°C increase in temperature.^22^–^24^ When the effects of hyperthermia and thermal tolerance were evaluated in the normal human fibroblast cell lines, Rassphorst and Azzam^25^ observed that fibroblasts developed thermal tolerance without heat damage. Less than 10% cell death was recorded after 6 to 8 hours of intracellular heating at temperatures less than or equal to 43°C. Between the temperatures of 43 and 44°C, thermal tolerance developed after 2 hours of heating and lasted up to 6 hours. All these experiments were performed with water baths or microwave probes. It is postulated that cancer tissues are relatively hypoxic and acidic secondary to inefficient vascular channels.^25^ When exposed to thermal insult, this vascular insufficiency leads to impaired cooling in tumor tissue compared to normal tissue, increasing the sensitivity of cancer to thermal injury.^25^ The exact molecular mechanisms of how thermal injury selectively kills tumor cells are largely unknown.

Nanotechnology provides a novel tool for magnetic hyperthermia. Since the first studies by Gordon et al.,^26^ iron oxide-based nanoparticles with superior magnetic and functionalized surface properties have been used for magnetic hyperthermia in targeted cancer treatment.^27^,^28^ Ito and Kobayashi^31^ evaluated the efficacy of magnetic hyperthermia by using dextran-coated magnetic nanoparticles in animals with various tumors, including B16 mouse melanoma, MM46 mouse mammary carcinoma, PC3, and LNCaP human prostate cancer in athymic mice, spontaneously occurring primary melanoma in transgenic mice, T-9 rat glioma, rat prostate cancer PLS10, Os515 hamster osteosarcoma, VX-7 squamous cell carcinomas in rabbit tongue, and human breast cancer BT474 (HER2-positive) in nude mice. Direct injection of nanoparticles into the solid tumor followed by magnetic hyperthermia application led to complete tumor regression in 96% of animals. In a clinical trial, Maier-Hauff et al.^32^ performed intratumoral injection of iron-oxide nanoparticles in 59 patients with recurrent glioblastoma multiforme. Magnetic hyperthermia was coupled with a reduced dose of fractionated stereotactic radiotherapy (a median dose of 30 Gy). The median overall survival of patients was 13.4 months, which was considerably longer than the typical median survival of 6 months in patients undergoing conventional radiotherapy.

In this paper, we present the first evaluation of magnetic hyperthermia using dextran-coated iron nanoparticles in a retinoblastoma cell line. At 24 hours, one session of magnetic hyperthermia induced 46% to 73% of death in Y79 retinoblastoma cells, suggesting that apoptosis started less than 24 hours after magnetic induction (Fig. 1). Effective cell death was observed with 0.75 and 1 mg/ml of nanoparticle concentration at 24 and 72 hours after magnetic hyperthermia. We observed maximum tumor cytotoxicity at 24 hours. Decreased cell death at 72 hours may indicate that surviving tumor cells continue to proliferate, selecting for resistant cell types. There was minimal cytotoxicity in ARPE-19 cells after 24 and 72 hours of magnetic hyperthermia at all nanoparticle concentrations, demonstrating that magnetic hyperthermia selectively induces cell death in tumor cells in a dose- and time-dependent manner. Our data demonstrate that optimal nanoparticle concentration for tumor cytotoxicity, while having a minimal cytotoxic effect on nontumor cells, was 0.75 mg/ml. However, we acknowledge that there are inherent caveats of studying cytotoxic effects of magnetic hyperthermia in in vitro cell culture systems. The differences in cell culture media (10% FBS for ARPE-19 vs. 20% for Y79 cells) and morphologic changes that occur in ARPE-19 cells upon moving from adherent cultures to suspensions in cryotubes may influence the uptake of nanoparticles, which might affect cytotoxicity.

We observed that dextran-coated nanoparticles are engulfed in the cytoplasm, lysosomes, and endosomes of the ARPE-19 and Y79 cells. Overgaards^33^ evaluated the ultrastructural changes in mouse mammary carcinoma after hyperthermia. The authors first observed a decrease in cell size with an increase in the number and activity of cytoplasmic lysosomes and disaggregated polyribosomes in the first 6 hours. This was followed by cell shrinkage and reduction in the number of mitochondria within 6 to 24 hours and total disarrangement of cytoplasmic components in 24 hours. Magnetic nanoparticles induced ruffling of the cell surface, which resulted from internalization via micropinocytosis, forming endosomes.^34^–^36^ With magnetic hyperthermia, nanoparticles leaked into the cytoplasm and produced reactive oxidative species, which led to DNA and mitochondrial damage and protein oxidation. Fang et al.^37^ proposed that magnetic nanoparticles increase the activation of the phosphatidylinositol 3-kinase/Akt/Bad pathway in LOVO cells, leading to the expression of cytochrome c, caspase 9, and caspase 3 proteins. Using TEM, we observed magnetic nanoparticles that were internalized into Y79 retinoblastoma cells. Forty-eight hours after the application of magnetic hyperthermia, caspase 3/7 activity increased selectively in tumor cells, indicating the activation of the mitochondrial cell death pathway. We also observed significant changes in the expression of apoptotic genes, including those involved in FAS and TNF-α signaling. Our gene expression data demonstrated that many signaling pathways involved in cell death were significantly elevated in tumor cells specifically after magnetic hyperthermia. This global change in apoptotic and antiapoptotic gene expression suggests that internalization of nanoparticles and magnetic hyperthermia lead to catastrophic cell damage and kill Y79 cells via both extrinsic and intrinsic apoptotic pathways.

Death receptors, such as the FAS and TNF receptors (FASR, and TNF-R1), are membrane proteins capable of inducing apoptosis and belong to the TNF-R superfamily.^38^ Following magnetic hyperthermia, gene expression profiling showed that genes in FAS and TNF-α signaling pathways were highly activated in Y79 retinoblastoma cells, reaching several hundred folds for some genes, including FAS ligand and TNF. Tran et al.^39^ showed that exposure to mild heat shock (30 minutes at 42°C) rapidly activated FAS-mediated apoptosis in cancer lines, including Jurkat and HeLa cells. FAS activates caspase-3 not only by inducing the cleavage of the caspase zymogen to its active subunits but also by stimulating the denitrosylation of its active-site thiol.^40^,^41^ In nude mice carrying human glioma cells, Ito et al.^42^ showed that hyperthermia using magnetic nanoparticles induced cell death throughout the tumor area and increased TNF-α gene expression by 3-fold. Our results indicate that FAS and TNF-α pathways are the primary drivers of apoptosis after magnetic hyperthermia treatment in Y79 cells.

Our data demonstrated that DCION treatment had a minimal cytotoxic effect on Y79 or ARPE-19 cells in the absence of magnetic hyperthermia. We found that magnetic hyperthermia via iron nanoparticles was selectively cytotoxic to Y79 retinoblastoma cells, providing evidence for developing magnetic hyperthermia as a potential treatment for retinoblastoma. In addition, we did not detect any caspase activation with iron nanoparticles in the absence of magnetic treatment, demonstrating that hyperthermia is the primary mechanism of tumor toxicity in our model.

Dextran-coated nanoparticles and magnetic hyperthermia might have clinical applications for intraocular tumors. Because intraocular injection is a commonly-used procedure for many retinal diseases, dextran-coated nanoparticles can be easily delivered intravitreally by using existing techniques. Nanoparticle-mediated magnetic hyperthermia can be used as an adjuvant therapy to enhance the outcome of chemotherapy or radiotherapy for eliminating intraocular tumors. However, the penetration of intravitreally delivered dextran-coated nanoparticles into the retinal tissue and the choroid and potential ocular toxicity are unknown. Further studies, including those involving animal models of intraocular tumors, are needed to answer these questions. Modifying nanoparticles with fluorescent labels and staining retinoblastoma tissue with various organelle-specific stains in animal models will also be needed to determine the exact subcellular localization of nanoparticles in in vivo applications.

In summary, similar to other cancer cells, Y79 retinoblastoma cells are exquisitely sensitive to thermal damage. We demonstrate that magnetic hyperthermia using dextran-coated iron nanoparticles selectively kills retinoblastoma cells in a dose- and time-dependent manner in vitro, while sparing nontumor cells. Magnetic hyperthermia induces apoptotic cell death in Y79 cells primarily via the intrinsic pathway activated by FAS and TNF-α signaling. With the recent advent of intravitreal chemotherapeutic injections in the management of retinoblastoma, magnetic hyperthermia with dextran-coated iron nanoparticles may be a promising therapeutic option.

## Supplementary Material

Supplement 1

Supplement 2
