# Supplementary material for: Magnetic Hyperthermia in Y79 Retinoblastoma and ARPE-19 Retinal Epithelial Cells: Tumor Selective Apoptotic Activity of Iron Oxide Nanoparticle
Source: Transl Vis Sci Technol. 2019 Sep 27;8(5):18. doi: 10.1167/tvst.8.5.18 (PMC6779177; doi:10.1167/tvst.8.5.18)
Supplement: Supplement 1 [file tvst-08-04-43_s01.pdf]

Supplemental Table 1. Genes upregulated 24 hours after magnetic hyperthermia in Y-79 retinoblastoma cells treated with 1 mg/ml of dextran-coated iron oxide nanoparticles compared to untreated cells

Gene	Description	Fold Change
FASLG	Fas ligand (TNF superfamily, member 6)	1133.0533
TNF	Tumor necrosis factor	665.9792
IL10	Interleukin 10	646.273
BAG3	BCL2-associated athanogene 3	583.8012
CASP14	Caspase 14, apoptosis-related cysteine peptidase	317.2186
TNFRSF9	Tumor necrosis factor receptor superfamily, member 9	269.2246
PYCARD	PYD and CARD domain containing	265.5181
LTBR	Lymphotoxin beta receptor (TNFR superfamily, member 3)	258.2575
BCL2A1	BCL2-related protein A1	140.0049
LTA	Lymphotoxin alpha (TNF superfamily, member 1)	130.0269
BIRC3	Baculoviral IAP repeat containing 3	112.9338
CFLAR	CASP8 and FADD-like apoptosis regulator	97.4101
CD70	CD70 molecule	52.6855
CD40	CD40 molecule, TNF receptor superfamily member 5	47.9237
CASP10	Caspase 10, apoptosis-related cysteine peptidase	44.4055
BCL2L10	BCL2-like 10 (apoptosis facilitator)	38.037
CD40LG	CD40 ligand	37.1683
CASP5	Caspase 5, apoptosis-related cysteine peptidase	35.2447
TNFRSF1A	Tumor necrosis factor receptor superfamily, member 1A	32.9605
FAS	Fas (TNF receptor superfamily, member 6)	32.1334
CASP8	Caspase 8, apoptosis-related cysteine peptidase	31.8378
HRK	Harakiri, BCL2 interacting protein (contains only BH3 domain)	31.7643
CD27	CD27 molecule	25.0372
TNFSF10	Tumor necrosis factor (ligand) superfamily, member 10	24.7496
CASP4	Caspase 4, apoptosis-related cysteine peptidase	24.1285
BCL2L11	BCL2-like 11 (apoptosis facilitator)	22.5648
TNFSF8	Tumor necrosis factor (ligand) superfamily, member 8	20.196
TNFRSF25	Tumor necrosis factor receptor superfamily, member 25	17.2996
RIPK2	Receptor-interacting serine-threonine kinase 2	17.1009
CASP1	Caspase 1, apoptosis-related cysteine peptidase	15.736
TNFRSF10A	Tumor necrosis factor receptor superfamily, member 10a	15.3057
BIK	BCL2-interacting killer (apoptosis-inducing)	11.8433
TNFRSF1B	Tumor necrosis factor receptor superfamily, member 1B	11.5728
MCL1	Myeloid cell leukemia sequence 1 (BCL2-related)	10.0515
BAG1	BCL2-associated athanogene	9.982
TNFRSF11B	Tumor necrosis factor receptor superfamily, member 11b	9.982
TNFRSF21	Tumor necrosis factor receptor superfamily, member 21	9.5533
TP53BP2	Tumor protein p53 binding protein, 2	6.3614
BAD	BCL2-associated agonist of cell death	5.3864
BCL10	B-cell CLL/lymphoma 10	5.2756
CYCS	Cytochrome c, somatic	5.0374
NOL3	Nucleolar protein 3 (apoptosis repressor with CARD domain	4.9565
BRAF	V-raf murine sarcoma viral oncogene homolog B1	4.646
TRADD	TNFRSF1A-associated via death domain	4.4982
BIRC6	Baculoviral IAP repeat containing 6	4.2457
GADD45A	Growth arrest and DNA-damage-inducible, alpha	4.026

TRAF3	TNF receptor-associated factor 3	3.8442
BCL2	B-cell CLL/lymphoma 2	3.7825
CASP2	Caspase 2, apoptosis-related cysteine peptidase	3.4248
TNFRSF10B	Tumor necrosis factor receptor superfamily, member 10b	3.3854
TRAF2	TNF receptor-associated factor 2	3.24
APAF1	Apoptotic peptidase activating factor 1	3.188
CASP9	Caspase 9, apoptosis-related cysteine peptidase	3.1514
NOD1	Nucleotide-binding oligomerization domain containing 1	3.0511
BNIP2	BCL2/adenovirus E1B 19kDa interacting protein 2	2.8865
BIRC5	Baculoviral IAP repeat containing 5	2.8402
CIDEB	Cell death-inducing DFFA-like effector b	2.7625
CIDEA	Cell death-inducing DFFA-like effector a	2.2595
BNIP3	BCL2/adenovirus E1B 19kDa interacting protein 3	2.1825
ABL1	C-abl oncogene 1, non-receptor tyrosine kinase	2.1228
AKT1	V-akt murine thymoma viral oncogene homolog 1	2.0936
BAK1	BCL2-antagonist/killer 1	2.0553
NFKB1	Nuclear factor of kappa light polypeptide gene enhancer in B-cells 1	1.9088
DIABLO	Diablo, IAP-binding mitochondrial protein	1.7124
BAX	BCL2-associated X protein	1.6849
TP53	Tumor protein p53	1.6849
DFFA	DNA fragmentation factor, 45kDa, alpha polypeptide	1.477
NAIP	NLR family, apoptosis inhibitory protein	1.4634
IGF1R	Insulin-like growth factor 1 receptor	1.3877
FADD	Fas (TNFRSF6)-associated via death domain	1.2478
BNIP3L	BCL2/adenovirus E1B 19kDa interacting protein 3-like	1.2164
DAPK1	Death-associated protein kinase 1	1.1777
BFAR	Bifunctional apoptosis regulator	0.9858
CASP3	Caspase 3, apoptosis-related cysteine peptidase	0.8722
BCL2L2	BCL2-like 2	0.8309
BID	BH3 interacting domain death agonist	0.664
CASP7	Caspase 7, apoptosis-related cysteine peptidase	0.664
BCL2L1	BCL2-like 1	0.6625
CASP6	Caspase 6, apoptosis-related cysteine peptidase	0.6399
TP73	Tumor protein p73	0.5998
XIAP	X-linked inhibitor of apoptosis	0.5584
BIRC2	Baculoviral IAP repeat containing 2	0.4463
CRADD	CASP2 and RIPK1 domain containing adaptor with death domain	0.4013
AIFM1	Apoptosis-inducing factor, mitochondrion-associated, 1	0.3701
