# Supplementary material for: Magnetic Hyperthermia in Y79 Retinoblastoma and ARPE-19 Retinal Epithelial Cells: Tumor Selective Apoptotic Activity of Iron Oxide Nanoparticle
Source: Transl Vis Sci Technol. 2019 Sep 27;8(5):18. doi: 10.1167/tvst.8.5.18 (PMC6779177; doi:10.1167/tvst.8.5.18)
Supplement: Supplement 2 [file tvst-08-04-43_s02.pdf]

Supplemental Table 2. Genes upregulated 24 hours after magnetic hyperthermia in Y-79 retinoblastoma cells treated with 1 mg/ml of dextran-coated iron oxide nanoparticles compared to Y-79 retinoblastoma cells exposed to dextran coated iron oxide nanoparticles without magnetic field application

Gene	Description	Fold Change
TNF	Tumor necrosis factor	964.3592
FASLG	Fas ligand (TNF superfamily, member 6)	800.8011
BAG3	BCL2-associated athanogene 3	632.7203
IL10	Interleukin 10	543.3515
CASP14	Caspase 14, apoptosis-related cysteine peptidase	369.8034
PYCARD	PYD and CARD domain containing	157.6189
TNFRSF9	Tumor necrosis factor receptor superfamily, member 9	129.4063
LTA	Lymphotoxin alpha (TNF superfamily, member 1)	117.7604
LTBR	Lymphotoxin beta receptor (TNFR superfamily, member 3)	90.524
BIRC3	Baculoviral IAP repeat containing 3	83.6729
CD40LG	CD40 ligand	76.4886
CFLAR	CASP8 and FADD-like apoptosis regulator	70.8628
BCL2A1	BCL2-related protein A1	60.5563
BCL2L10	BCL2-like 10 (apoptosis facilitator)	53.2101
TNFRSF1A	Tumor necrosis factor receptor superfamily, member 1A	48.284
FAS	Fas (TNF receptor superfamily, member 6)	45.187
CD40	CD40 molecule, TNF receptor superfamily member 5	42.9574
RIPK2	Receptor-interacting serine-threonine kinase 2	37.947
CASP10	Caspase 10, apoptosis-related cysteine peptidase	37.6304
CD70	CD70 molecule	34.8205
TNFSF8	Tumor necrosis factor (ligand) superfamily, member 8	34.6293
CASP4	Caspase 4, apoptosis-related cysteine peptidase	34.1119
CD27	CD27 molecule	33.7515
HRK	Harakiri, BCL2 interacting protein (contains only BH3 domain)	26.8848
TNFRSF10A	Tumor necrosis factor receptor superfamily, member 10a	21.4137
CASP8	Caspase 8, apoptosis-related cysteine peptidase	16.8447
BCL2L11	BCL2-like 11 (apoptosis facilitator)	15.9246
CASP1	Caspase 1, apoptosis-related cysteine peptidase	14.8938
TNFSF10	Tumor necrosis factor (ligand) superfamily, member 10	13.1983
BIRC5	Baculoviral IAP repeat containing 5	11.6475
TNFRSF11B	Tumor necrosis factor receptor superfamily, member 11b	9.5875
TNFRSF25	Tumor necrosis factor receptor superfamily, member 25	9.0375
BAG1	BCL2-associated athanogene	8.314
GADD45A	Growth arrest and DNA-damage-inducible, alpha	6.6848
MCL1	Myeloid cell leukemia sequence 1 (BCL2-related)	6.2819
BRAF	V-raf murine sarcoma viral oncogene homolog B1	6.0789
CASP5	Caspase 5, apoptosis-related cysteine peptidase	5.82
BIK	BCL2-interacting killer (apoptosis-inducing)	5.4483
TRADD	TNFRSF1A-associated via death domain	5.2232
TP53BP2	Tumor protein p53 binding protein, 2	5.1312
TNFRSF1B	Tumor necrosis factor receptor superfamily, member 1B	4.6483
CYCS	Cytochrome c, somatic	4.2441
BIRC6	Baculoviral IAP repeat containing 6	4.0156
BCL10	B-cell CLL/lymphoma 10	3.3422
APAF1	Apoptotic peptidase activating factor 1	3.3052
BCL2	B-cell CLL/lymphoma 2	2.9535

NOL3	Nucleolar protein 3 (apoptosis repressor with CARD domain	2.8915
CASP9	Caspase 9, apoptosis-related cysteine peptidase	2.833
CIDEA	Cell death-inducing DFFA-like effector a	2.5084
BNIP2	BCL2/adenovirus E1B 19kDa interacting protein 2	2.4759
TNFRSF10B	Tumor necrosis factor receptor superfamily, member 10b	2.3228
TP73	Tumor protein p73	2.2843
CASP2	Caspase 2, apoptosis-related cysteine peptidase	2.2362
TRAF3	TNF receptor-associated factor 3	2.1716
BAD	BCL2-associated agonist of cell death	1.9107
TRAF2	TNF receptor-associated factor 2	1.7993
BNIP3	BCL2/adenovirus E1B 19kDa interacting protein 3	1.7604
CIDEB	Cell death-inducing DFFA-like effector b	1.7065
BNIP3L	BCL2/adenovirus E1B 19kDa interacting protein 3	1.6161
NFKB1	Nuclear factor of kappa light polypeptide gene enhancer in B-cells 1	1.6092
NOD1	Nucleotide-binding oligomerization domain containing 1	1.4861
XIAP	X-linked inhibitor of apoptosis	1.4839
DIABLO	Diablo, IAP-binding mitochondrial protein	1.4131
NAIP	NLR family, apoptosis inhibitory protein	1.3926
FADD	Fas (TNFRSF6)-associated via death domain	1.3786
BIRC2	Baculoviral IAP repeat containing 2	1.3668
ABL1	C-abl oncogene 1, non-receptor tyrosine kinase	1.3587
BAK1	BCL2-antagonist/killer 1	1.3039
DFFA	DNA fragmentation factor, 45kDa, alpha polypeptide	1.1488
BCL2L2	BCL2-like 2	1.1406
IGF1R	Insulin-like growth factor 1 receptor	1.0844
AKT1	V-akt murine thymoma viral oncogene homolog 1	1.0669
DAPK1	Death-associated protein kinase 1	1.0255
TNFRSF21	Tumor necrosis factor receptor superfamily, member 21	0.9208
TP53	Tumor protein p53	0.907
BFAR	Bifunctional apoptosis regulator	0.8538
CASP3	Caspase 3, apoptosis-related cysteine peptidase	0.7628
BAX	BCL2-associated X protein	0.7226
CASP7	Caspase 7, apoptosis-related cysteine peptidase	0.5557
CRADD	CASP2 and RIPK1 domain containing adaptor with death domain	0.5418
AIFM1	Apoptosis-inducing factor, mitochondrion-associated, 1	0.5398
BID	BH3 interacting domain death agonist	0.5237
CASP6	Caspase 6, apoptosis-related cysteine peptidase	0.4953
BCL2L1	BCL2-like 1	0.4689
